# Editorial: Pediatric Pulmonary Hypertension

**DOI:** 10.3389/fped.2015.00105

**Published:** 2015-11-30

**Authors:** Maurice Beghetti, Frederic Lador

**Affiliations:** ^1^Pediatric Cardiology Unit, Children’s University Hospital, University of Geneva, Geneva, Switzerland; ^2^Pulmonary Hypertension Program, University of Geneva, Geneva, Switzerland; ^3^Division of Pneumology, University of Geneva, Geneva, Switzerland

**Keywords:** pediatrics, pulmonary circulation, pulmonary hypertension, diagnostic tests, routine, therapeutics

Pulmonary hypertension (PH) can present at any age from the neonatal period to adulthood. Pediatric PH is similar to adults but differentiates itself as in a growing child the lungs are still developing (1). It is a hemodynamic condition defined by an increased pulmonary artery mean pressure above or equal to 25 mmHg (1). This can be associated with multiple clinical conditions forming different etiological groups with specific and diverse pathological and pathobiological characteristics (1). When implying an abnormal reduction of small pulmonary arteries section due to endothelial dysfunction, medial proliferation, and adventitial fibrosis, the disease is called pulmonary arterial hypertension (PAH). PAH induces typically a severe and devastating PH that leads to right heart failure and premature death.

Until recently, the vast majority of studies published on PH were in the adult population but, as highlighted by the series of manuscript published in this issue of frontiers in pediatrics, awareness about pediatric PH is growing. In this line, a specific subgroup was dedicated to pediatrics at the last world symposium on PH with the publication of an excellent summary (1). Manuscripts not only on clinical research but also on basic science further highlight the interest in pediatric PH.

Epidemiologic data, mainly sourced from national and international registries, have recently been published, but the content of these registries varies depending on the center involved and the inclusion criteria (2). Indeed, most of the registries, including centers from developed countries, do not report children with PAH associated with HIV, in contrast to reports coming from developing countries (3). This may be due to the absence of this problem in developed countries in association with aggressive antiretroviral therapies or the absence of screening. L’Huillier et al. report the data from a Swiss cohort of patients with HIV (4). No patient presented with PH, but screening of PH was not systematically performed. This must be kept in mind when analyzing epidemiologic data.

In pediatrics, there is a need and a constant search for non-invasive assessments of PH, mainly because right heart catheterization, whenever it is still considered as the gold standard for hemodynamic evaluation and PH diagnosis is associated with potential risk in this particular population. So, the development of non-invasive tools for hemodynamic evaluation is not only desirable but mandatory in pediatrics. To this purpose, echocardiography gives invaluable information during the follow-up. Jone and Ivy did an excellent review of the current knowledge application of this approach in pediatric PH (5) and offer a basis for the remaining work dedicated to validation of most of the discussed parameters. Similarly, Day et al. reported their experience with the use of oxygen and sildenafil for assessment of pulmonary vascular reactivity in children (6). The response to vasoreactivity testing is crucial for orientation of treatment strategy as responders can be treated with calcium channel blockers with an excellent survival rate. This was clearly demonstrated in adults (7) but we still have to work to define a similar approach in children. Here, a major question still remains: what is the definition of a responder? The manuscript of Day et al. brings some more information with regards to vasoreactivity assessments in pediatric PH.

Still in the field of non-invasive assessment and potential markers of disease, Latus et al. reported their experience with the analysis of heart rate variability (HRV) showing that this parameter may be altered in pediatric patients with PH (8). HRV may indeed be related to disease severity and serve as an additional marker for PH evaluation.

Although there is still no cure for PAH, quality of life and survival have been improved significantly with specific drug therapies (9). Nevertheless, management of pediatric PAH remains challenging and depends mainly on results from adult clinical trials, a few specific pediatric studies and pediatric experts’ recommendations. Any additional report of the use of targeted therapies in children is of interest. Siehr et al. reported their experience with the use of sildenafil and concentrated on its potential side effects (10). One must remember that a question was raised on the use of sildenafil in children as the STARTS study reported an increased risk of death using high doses (11, 12). This led to a warning from the FDA and the approval of the medium dose by the European Medical Authorities. Even, if Siehr et al. reported no new side effects, they mention that the incidence of vascular, gastrointestinal, and neurologic side effects in pediatric patients on sildenafil therapy was 30%. Side effects were more common in patients on combination therapy with an endothelin receptor antagonist and/or a prostacyclin than in patients on sildenafil monotherapy.

Prior to the development of new therapeutic pathways, recognition of all pathobiological pathways involved in the development of pulmonary vascular disease are needed. Van loon et al. (13) reported their experience with erythropoietin (EPO) (10). In experimental PAH, EPO treatment restored the number of circulating endothelial progenitor cells (EPCs) to control level, improved pulmonary vascular remodeling, and showed important interplay with heme oxygenase (HO) activity. Inhibition of increased HO activity in PAH rats exacerbated progression of pulmonary vascular remodeling, despite the presence of restored number of circulating EPCs. It may be that both EPO-induced HO and EPCs are promising targets to improve the pulmonary vasculature in PAH.

Different specific issues related to pediatric PAH have been reported and highlight the need to continue active research in this field. It is of paramount importance to better understand the pathobiology of the disease, its behavior and discover new treatment pathway in order to finally curing this devastating disease.

## Author Contributions

MB wrote the editorial and reviewed most of the research topic manuscript. FL reviewed the content and added own ideas in the editorial; he also read all research topic manuscripts.

## Conflict of Interest Statement

The authors declare that the research was conducted in the absence of any commercial or financial relationships that could be construed as a potential conflict of interest.
